# Draft Genome Sequence of *Okeania* sp. Strain KiyG1, Assembled from Single-Amplified Genomes Collected from Cape Kiyan, Okinawa, Japan

**DOI:** 10.1128/MRA.00837-20

**Published:** 2020-11-12

**Authors:** Muhammad Wahyudin Lewaru, Yohei Nishikawa, Keigo Ide, Masato Kogawa, Masahito Hosokawa, Ashok Zachariah Samuel, Shinpei Sumimoto, Handung Nuryadi, Shoichiro Suda, Haruko Takeyama

**Affiliations:** a Department of Life Science and Medical Bioscience, Waseda University, Tokyo, Japan; b Computational Bio Big-Data Open Innovation Laboratory, National Institute of Advanced Industrial Science and Technology, Tokyo, Japan; c Research Organization for Nano and Life Innovation, Waseda University, Tokyo, Japan; d Faculty of Science, University of the Ryukyus, Okinawa, Japan; e Institute for Advanced Research of Biosystem Dynamics, Waseda Research Institute for Science and Engineering, Tokyo, Japan; Georgia Institute of Technology

## Abstract

The genus *Okeania* is a globally distributed group of microorganisms that live in shallow seabed regions. These organisms play several environmentally important roles and are also known producers of several active secondary metabolites with potential human applications. Here, we present a draft genome of *Okeania* sp. strain KiyG1 (92.7% completeness) that was assembled from four single-amplified genomes.

## ANNOUNCEMENT

Cyanobacteria play several important ecological roles in marine ecosystems, particularly contributing to nitrogen fixation and reduction of global carbon flux (1). They are also known producers of several biologically active secondary metabolites (2). However, their genomic details remain incomplete. The filamentous cyanobacteria, including *Okeania* spp. (defined in 2013), usually form biological mats composed of various kinds of bacteria (3), often with relatively lower abundance of the target cyanobacteria, which causes DNA contamination and reduces the accuracy of genome sequencing (4, 5). Therefore, determining the genome sequence from isolated single cells is advantageous (6, 7).

Cyanobacteria of *Okeania* sp. strain KiyG1 (GenBank accession number LC534992) were collected from the shallow seabed (water depth of 10 cm) of Cape Kiyan in Okinawa, Japan (26°04′52.6″N, 127°39′25.5″E), on 15 June 2019. The samples were washed with artificial seawater, cut into small pieces with a scalpel, and then treated with 5% sodium dodecyl sulfate (Sigma-Aldrich, St. Louis, MO, USA) in Dulbecco's phosphate-buffered saline (DPBS) (Thermo Fisher Scientific, Waltham, MA, USA) for about 5 min at room temperature. This mixture was then filtered through a membrane filter (MF-Millipore filter with 5-μm pore size, SMWP04700; Millipore, Merck, Germany), and the filamentous cyanobacteria trapped on the membrane were transferred into 1 ml DPBS. The mixture was then vortex-mixed to disentangle the cyanobacteria to single cells. Eight single cells were picked from the vortex-mixed sample by pipetting (Drummond Scientific, Broomall, PA, USA) during observation through a microscope (CKX53, with magnification of ×10; Olympus, Tokyo, Japan). The cells were then transferred to a 384-well plate containing 0.8 μl DPBS and were physically ground using a micropipette tip (123R-254CS; Fukae Kasei Co., Japan). The whole genome in the resulting samples was amplified with the REPLI-g single-cell kit (Qiagen Inc., Valencia, CA, USA) at 30°C for 120 min. Sufficient DNA yield (>1 ng) for sequencing was available for four samples. The independent sequencing libraries were prepared from each of the four samples with the Nextera XT DNA library preparation kit (Illumina, San Diego, CA, USA) according to the manufacturer’s instructions and were sequenced on an Illumina MiSeq system for 75 cycles of paired-end sequencing, generating 116 to 145 Mbp. Low-quality reads (≥50% of bases with quality scores of <25) were eliminated, and the 3′ ends (quality scores of <20) were trimmed using FASTX-Toolkit (http://hannonlab.cshl.edu/fastx_toolkit) and PRINSEQ (8). The cleaned reads (>20 bp) were assembled using SPAdes v3.13.1 (9) with the following settings: careful, sc. We successfully acquired four single-cell genomes. Because the sequences of >100 single-copy marker genes identified with CheckM v1.0.18 (10) exhibited >99.99% identity, DNA from the four amplified genomes was mixed evenly and sequenced with a GridION system (Oxford Nanopore Technologies Ltd., Oxford, UK) using the rapid sequencing kit (SQK-RAD004; Oxford Nanopore Technologies Ltd.), generating 1.13 Gbp. The long-read draft genome was assembled using Flye v2.7.1 (11) and polished with Pilon v1.22 (12).

The polished long-read draft genome of *Okeania* sp. strain KiyG1 was evaluated with QUAST v5.0.2 (13) and CheckM v1.0.18 and annotated with Prokka v1.13 (14). The genome completeness was 92.74%, and the contamination was 2.91%. Additional genome statistics for KiyG1 are provided in Table 1. Default parameters were used for all software unless otherwise noted.

**TABLE 1:** Assembly and quality statistics for the *Okeania* sp. strain KiyG1 single-amplified genome

Assembly statistic	Result for *Okeania* sp. strain KiyG1
No. of contigs	198
Total contig length (bp)^*a*^	10,982,302
*N*_50_ (bp)^*a*^	79,819
GC content (%)^*a*^	36.81
Completeness (%)^*b*^	92.74
Contamination (%)^*b*^	2.91
Predicted genome size (bp)^*b*^	11,842,034
No. of coding sequences^*c*^	9,096
No. of tRNA genes^*c*^	33

a Estimated with QUAST.

b Estimated with CheckM.

c Estimated with Prokka.

### Data availability.

The assembled genomes reported in this article were deposited in DDBJ under the accession number SAMD00236478. The raw reads are also available, under the accession numbers SAMD00233784 to SAMD00233787 (for Illumina reads) and SAMD00233788 (for Nanopore reads).
